# Current state of phoenixin—the implications of the pleiotropic peptide in stress and its potential as a therapeutic target

**DOI:** 10.3389/fphar.2023.1076800

**Published:** 2023-02-13

**Authors:** T. Friedrich, A. Stengel

**Affiliations:** ^1^ Charité Center for Internal Medicine and Dermatology, Department for Psychosomatic Medicine, Charité—Universitätsmedizin Berlin, corporate member of Freie Universität Berlin, Humboldt-Universität zu Berlin and Berlin Institute of Health, Berlin, Germany; ^2^ Department of Psychosomatic Medicine and Psychotherapy, University Hospital Tübingen, Tübingen, Germany

**Keywords:** anxiety, gut-brain, depression, psychosomatic, phoenixin, nesfatin, stress, anorexia

## Abstract

Phoenixin is a pleiotropic peptide, whose known functions have broadened significantly over the last decade. Initially first described as a reproductive peptide in 2013, phoenixin is now recognized as being implicated in hypertension, neuroinflammation, pruritus, food intake, anxiety as well as stress. Due to its wide field of involvement, an interaction with physiological as well as psychological control loops has been speculated. It has shown to be both able to actively reduce anxiety as well as being influenced by external stressors. Initial rodent models have shown that central administration of phoenixin alters the behavior of the subjects when confronted with stress-inducing situations, proposing an interaction with the perception and processing of stress and anxiety. Although the research on phoenixin is still in its infancy, there are several promising insights into its functionality, which might prove to be of value in the pharmacological treatment of several psychiatric and psychosomatic illnesses such as anorexia nervosa, post-traumatic stress disorder as well as the increasingly prevalent stress-related illnesses of burnout and depression. In this review, we aim to provide an overview of the current state of knowledge of phoenixin, its interactions with physiological processes as well as focus on the recent developments in stress response and the possible novel treatment options this might entail.

## 1 Introduction

Phoenixin was first described as a reproductive peptide in 2013 by Yosten et al. (Yosten et al., 2013) who employed a bioinformatic approach in order to deduce possible conserved peptides from human genome data (Yosten et al., 2013). Its highly conserved nature is shown by the very small inter-species variability with no differences in the phoenixin-14 sequence in mammals and only one amino acid variation in the phoenixin-20 sequence in the initially studied tissues (Yosten et al., 2013; Jiang et al., 2015a). Since its discovery, a total of 69 original research papers (10/22) were published on Pubmed. The majority of these research papers have focused on reproduction as well as the neurophysiology of phoenixin, with several publications also focusing on food/water intake and digestion as well as its psychopathological properties and inflammation. Other publications have studied memory impairment and cardiovascular implications.

Several variants of phoenixin have been described, namely 5, 8, 14, 15, 17, 20, 26, 36, and 42 amino-acid long variants (Yosten et al., 2013; Private Communication; Cowan et al., 2015; Phoenix Pharmaceuticals, INC 2021; Pubmed, 2022). The physiologically relevant peptides and targets of most studies are phoenixin-14 and phoenixin-20 (Yosten et al., 2013; Pubmed, 2022). Both forms show a similar affinity to pituitary cell membranes in cell cultures (Yosten et al., 2013). Its precursor protein SMIM20 (also known as MITRAC7 and C4orf52) (Prinz et al., 2017; National Library of Medicine, 2022a) is scarcely researched so far. It is part of the **MI**tochondrial **T**ranslation **R**egulation **A**ssembly intermediate of **C**ytochrome c (MITRAC), which regulates parts of the respiratory chain, namely the abundance of cytochrome c oxidase (complex IV) through stabilization of COX1, a part of cytochrome c oxidase biogenesis (Dennerlein et al., 2015; Bourens and Barrientos, 2017). This is part of a feedback loop to avoid mitochondrial damage due to excessive precursor buildup (Dennerlein et al., 2015). Deviations from normal complex IV functioning can be the cause of encephalo-cardiomyopathies (Bourens and Barrientos, 2017). Although its absence does not seem to lead to a complete dysfunction of complex IV, early studies have shown a 50% reduction in complex IV activity (Bourens and Barrientos, 2017). Phoenixin-14 is MITRAC7’s most abundant cleavage product, potentially a result of carboxypeptidase cleavage (Lyu et al., 2013).

This review aims to summarize the currently available literature concerning phoenixin, its receptor and its involvement in physiology and pathology as well as outline potential pharmaceutical targets.

## 2 State of knowledge

### 2.1 Sites of expression—neuroanatomy and neurophysiology

Phoenixin peptide is mainly found in the hypothalamus, but to a lesser extent also present in heart, thymus, gastrointestinal tract, spleen and pancreas (Yosten et al., 2013; Prinz et al., 2017). Phoenixin mRNA was detected in various brain regions, most abundantly in the hypothalamus, amygdala and cerebellum but also the pituitary and brainstem as well as the adrenal gland, heart, ovary, uterus, and kidney (Yosten et al., 2013). Phoenixin immunoreactivity in the brain was mostly observed in the bed nucleus of the stria terminalis (BST), the paraventricular nucleus (PVN), the supraoptic nucleus (SON), lateral hypothalamus, the central amygdaloid nucleus (CeM), the arcuate nucleus (Arc), ventromedial hypothalamus, median eminence, zona incerta, perifornical area, dorsal hypothalamus, the spinocerebellar tract ventral (vsc) and dorsal (dsc), substantia nigra reticulata, the Edinger-Westphal nucleus (EW), raphe pallidus (RPa), the spinal trigeminal tract (sp5), as well as the nucleus of the solitary tract (medial division; mNTS), dorsal motor nucleus of vagus (DMN) and area postrema (AP) (Yosten et al., 2013; Prinz et al., 2017). It has also been observed in the anterior and posterior lobe of the pituitary giving rise to a – besides endocrine signaling – possible paracrine mode of action (Yosten et al., 2013). Phoenixin immunoreactivity is also present in the spinal cord of rodents, namely in the superficial dorsal horn, spinal trigeminal tract as well as dorsal root and trigeminal and nodose ganglion cells (Lyu et al., 2013). Interestingly, phoenixin seems to be distinctly present in cell processes, while phoenixin immunoreactive neurons – when present – seem to be mostly smaller neurons (Lyu et al., 2013; Yosten et al., 2013).

### 2.2 Receptor

While not definitively proven, GPR173 is the most commonly assumed receptor of phoenixin. The idea of an orphan GPR as phoenixin’s receptor was initially introduced by Lyu et al., since phoenixin was discovered employing a search for ligands for orphan GPRs (Lyu et al., 2013). Treen et al. then suggested GPR173, also known as superconserved receptor expressed in the brain 3 (SREB3), which is an orphan GPR, as the phoenixin receptor (Treen et al., 2016). It is mostly expressed in the brain and ovaries (Matsumoto et al., 2000), overlapping with phoenixin’s known functions in reproduction, anxiolysis and orexigenic effects (Friedrich and Stengel, 2021). More specifically, GPR173 is most prominently present in the piriform cortex, lateral septum (LS), BST, anteroventral periventricular nucleus (AVPN), medial preoptic area (MPO), PVN, ventromedial hypothalamus (VMH), hippocampus, SON, lateral hypothalamic area, paraventricular nucleus of the thalamus, dorsomedial nucleus of the hypothalamus (DMH), CeM and the ventral premammillary nucleus (PMV) (Stein et al., 2016). One publication predating the discorvery of phoenixin found GPR173 mRNA in several tissues extracted from C57BL/6 mice: Medium expression relative to control was found in the hypothalamus, cerebellum, olfactory bulb, hippocampus and striatum, low expression was observed in the cerebral cortex, pituitary, brainstem, retina, eye, ovary, uterus, vena cava, aorta, heart atrium and thyroid (descending order of expression) (Regard et al., 2008). In addition, GPR173 mRNA was also detected in the 3T3-L1 preadipocyte cell line as well as rat primary adipocytes (Billert et al., 2018). GPR173s genetic information is located on the X chromosome, speculatively explaining the gender-specific effects of phoenixin (National Library of Medicine, 2022b). Similar to GnRH and its receptor, phoenixin-20 regulates the expression of GPR173 (Treen et al., 2016; Yang et al., 2020). Curiously, while phoenixin-20 led to an initial increase in GPR173, another study observed a blunted effect of phoenixin-20 after pretreatment with phoenixin-20, leading to speculations about a possible desensitization and suggesting a negative feedback loop after longer exposure of GPR173 to its proposed ligand (Treen et al., 2016). Knockdown of GPR173 with siRNA ameliorated phoenixin-20’s influence on kisspeptin and GnRH regulation (Treen et al., 2016) as well as the observed increase of gonadotropin-releasing hormone (GnRH)-dependent luteinizing hormone (LH) release *in vitro*, and significantly prolonged the estrous cycle in rodents (Stein et al., 2016). In addition, it reduced a phoenixin-20-induced vasopressin release (Gasparini et al., 2018). *In vitro*, GPR173 expression in pituitary cell cultures was disrupted by both bisphenol A and the fatty-acid palmitate (McIlwraith et al., 2019), the latter notably was previously shown to increase phoenixin expression (McIlwraith et al., 2018). Interestingly, this palmitate induced reduction in GPR173 expression was only affected in female but not male pituitary cells (McIlwraith et al., 2019), suggesting a gender-specific role of phoenixin.

The initial report suggested that downstream of the receptor, GPR173 signaling could then activate the cAMP-PKA pathway leading to the phosphorylation of the transcription factor CREB (Treen et al., 2016). Notably, MAPK and PKC were seemingly not involved in phoenixin-20’s modulation of GnRH expression (Treen et al., 2016). It should be noted that GnRH_(1–5)_ also activates GPR173 (Wu et al., 2005; Larco et al., 2013a; Treen et al., 2016). Interestingly, GnRH_(1–5)_ does not seem to lead to a coupling of GPR173 with a G-alpha subunit, but rather employ β-arrestin 2 to exert its effect in immortalized GnRH neurons (Larco et al., 2013b). GPR173 activation through GnRH metabolites should therefore be considered a major confounding factor in studies on GPR173 (Treen et al., 2016).

Recently the receptor-ligand relationship of phoenixin and GPR173 has, however, been called into question (Yanez-Guerra et al., 2022). Since the genomic information of GPR173 is not equally present throughout the evolutionary steps of species as the genomic information of phoenixin precursors, the authors suggest that a different receptor must exist (Yanez-Guerra et al., 2022). In addition, calcium mobilization essays showed no increased activation of any of the three SREBs in the presence of phoenixin, when GPR173 was coupled with either $G_{qi9\;}or\;G_{qs5}$ (Yanez-Guerra et al., 2022). The authors of this recent study however also state, that there are cases in which this method delivered false results. In addition, since GnRH_(1–5)_ induces signaling through β-arrestin 2 (Larco et al., 2013b), this could also be the case for phoenixin. Although this is the only paper so far regarding GPR173 as an unlikely receptor for phoenixin, it should be mentioned especially in the absence of any direct proof that phoenixin actually does bind to the GPR173. Interestingly, several inverse GPR173 agonists with cross-reactivity to GPR85 and GPR27 were described previously, but have not yet been used in any of the studies regarding GPR173 and phoenixin (Yanai et al., 2016). Locations of GPR173 and phoenixin are summarized in supplementary table 1.

### 2.3 Co-expression with nesfatin-1

Early in the process of better understanding phoenixin’s properties, a co-expression with the peptide nesfatin-1 was described (Palasz et al., 2015). This is especially interesting in light of its opposing physiological effects, that became more and more apparent after further research on phoenixin. These properties of nesfatin-1 include an anorexigenic effect (Atsuchi et al., 2010) and anxiety-increasing properties (Merali et al., 2008), which are in opposition to phoenixin-14’s orexigenic (Schalla et al., 2017) and anxiolytic (Jiang et al., 2015a) effects. Considering their extensive co-expression of 70%–86% in the hypothalamus (Palasz et al., 2015), a functional relationship seems possible. This co-expression was highest in the Arc with a co-expression of 86% (Palasz et al., 2015).

Nesfatin-1 is also involved in glucose and energy homeostasis (Foo et al., 2010; Stengel and Taché, 2013), both processes speculated to be also a target of phoenixin signaling (Friedrich and Stengel, 2021). Nesfatin-1 is itself highly co-expressed with vasopressin, oxytocin, proopiomelanocortin (POMC)/cocaine and amphetamine-regulated transcript (CART) and neuropeptide Y (NPY) (Goebel-Stengel and Wang, 2013; Palasz et al., 2015). These neuropeptides are key elements of hunger/appetite and thereby energy homeostasis (Matafome et al., 2017) as well as water/electrolyte homeostasis (Bichet, 2018). Nesfatin-1 signaling targets these neuropeptides in the PVN as well as the Arc (Palasz et al., 2015), the ladder being an important nucleus of food intake regulation (Schwartz et al., 2000) and as mentioned above also the nucleus with the highest overlap in expression of nesfatin-1 and phoenixin (Palasz et al., 2015). This overlap falls in line with the numerously observed effects of phoenixin on food intake and suggests a possible interaction in this regard. It should be mentioned that the observed overlap in the respective effects could potentially stem from their similar pattern of expression without necessarily proving a direct interaction or feedback loop.

### 2.4 Effects and involvement

#### 2.4.1 Reproduction

Reproduction is the initially described field of influence of phoenixin-20, which modulates the expression of GnRH receptor in the pituitary (Yosten et al., 2013). In the initial study, the authors speculate that the expression of phoenixin could potentially be modulated by estrogen and be part of the preovulatory release of LH and FSH (Yosten et al., 2013). Phoenixin-14 and -20 increased the GnRH-stimulated release of LH and to a lesser extent follicle-stimulating hormone (FSH) mRNA levels *in-vitro* (Yosten et al., 2013). This effect is probably gender specific, as phoenixin-20 did not alter LH release in male but only female pituitary cell cultures (Yosten et al., 2013). Another study found both phoenixin-14 and -20 to increase growth hormone (GH) and the GH-releasing hormone receptor (GHRH-R) expression in pituitary cell cultures of spotted scats as well as *in vivo* (Wang et al., 2018). Phoenixin-20 also potentiated the effects of GnRH on LH release in cell cultures, which was dependent on its putative receptor GPR173 (Stein et al., 2016).

LH release was also affected *in vivo*, with a significant increase in plasma LH levels after intracerebroventricular (icv) injection of phoenixin-20 (Stein et al., 2016). Since GnRH modulation can be autoregulated (Kaiser et al., 1993; Lerrant et al., 1995), phoenixin-20 leads to an increase of GnRH-induced GnRH receptor upregulation, thereby supporting its feedback loop (Yosten et al., 2013). Interestingly, icv administration of phoenixin siRNA led to a prolongation of the estrous cycle in rats, showing its physiological necessity for the estrous cycle in rodents (Yosten et al., 2013).

The influence on the estrous cycle might also be a result of a modulated kisspeptin signaling, since phoenixin-20 increased Kiss1 mRNA expression *in vitro* (Treen et al., 2016). Plasma phoenixin-14 is also elevated in women with polycystic ovary syndrome (PCOS) and correlates to the LH levels in those patients (Ullah et al., 2017). Patients suffering from PCOS exhibited a negative correlation between phoenixin-14 and estrogen as well as a positive correlation between phoenixin-14 and nesfatin-1 (Ullah et al., 2017). Interestingly, when treated with the endocrine disruptive molecule BPA only male hypothalamic cell lines showed a decreased phoenixin expression (McIlwraith et al., 2018), indicating a sex-specific regulatory mechanism. With regard to reduced fertility, phoenixin-14 was also shown to have potential pharmaceutical properties, since a recent study in rodents suggests a possible use in obesity-associated reduced fertility, where oral application of phoenixin-14 reduced serum insulin, testosterone, reactive oxygen species (ROS), malondialdehyde (MDA), tumor-necrosis-factor-α (TNF-α) and caspase-3 while increasing estrogen, progesterone, LH and FSH (Basha et al., 2022).

Interestingly, both an antagonism of the GnRH receptor with the drug cetrorelix as well as treatment with buserelin, an agonist of this receptor, led to an increase in hypothalamic expression of phoenixin’s precursor SMIM20 (Suszka-Switek et al., 2019). It is to note, that cetrorelix induces a plethora of effects, including anxiolytic, anti-depressant and memory-enhancing effects (Telegdy et al., 2009; Suszka-Switek et al., 2019) and its interaction with GPR173 is not yet elucidated. A similar effect was observed in the pituitary and ovary of rats, which also exhibited an increased expression of SMIM20 after both agonism and antagonism of GnRH-R (Suszka-Switek et al., 2019). Contrary to SMIM20, GPR173 was downregulated in the hypothalamus and pituitary after both agonism and antagonism of GnRH-R (Suszka-Switek et al., 2019), further supporting a possible feedback loop with phoenixin/SMIM20. Phoenixin has also been shown to enhance follicular growth *via* GPR173 activation, which was suppressed by GPR173 siRNA (Nguyen et al., 2019). In another study, phoenixin-14 as well as nesfatin-1 administered icv in male rats did not alter GnRH plasma levels, while leading to an increase in plasma levels of FSH, LH and testosterone (Guvenc et al., 2019). Their effects were most pronounced 30 min after injection and even more so when administered together, suggesting a synergistic effect of phoenixin-14 and nesfatin-1 in male reproduction (Guvenc et al., 2019). Since phoenixin serum levels of patients suffering from endometriosis were lower than those of healthy women, the phoenixin pathway was also proposed as a possible target for endometriosis treatment (Kulinska et al., 2021).

#### 2.4.2 Pain and pruritus

Phoenixin has shown analgetic properties when injected intrathecally, specifically a suppression of visceral pain stimuli when the amidated phoenixin was injected, while thermal pain was unaffected (Lyu et al., 2013). Episodes of visceral pain as part of epileptiform activity have been reported in one case report of a family with a deletion in chromosome 4p15, which is the genetic location of phoenixin’s precursor protein SMIM20 (Kinton et al., 2002). Although this does not necessarily indicate an implication of phoenixin, both its isoforms were shown to exert a pruritogen effect in mice, that was reversible by kappa-opioid receptor agonist application together with phoenixin-14 (Cowan et al., 2015). Since pruritus is hypothesized to be a mild pain sensation in the context of neuronal signaling, this would further support an involvement of phoenixin in pain modulation (Cowan et al., 2015). Neurons that exhibited phoenixin immunoreactivity were also shown to have axons reaching the *epidermis* (Cowan et al., 2015), leading to the assumption that phoenixin might not only play a role in visceral pain, but also an involvement in peripheral sensation. Notably, the pruritogen effect was most likely not mediated centrally, but locally, since this is one of the few publications showing an effect of phoenixin after peripheral application (Cowan et al., 2015).

#### 2.4.3 Cardiac physiology

Although not extensively studied with regard to cardiac performance, phoenixin-14 was shown to reduce contractility and relaxation of perfused rat hearts and seems to exert cardioprotective effects *via* the survivor activating factor enhancement (SAFE) and reperfusion injury salvage kinase (RISK) pathways as well as *via* influencing mitochondrial ATP-dependent potassium (Mito-KATP) channels (Rocca et al., 2018). These antiapoptotic properties of phoenixin-14 were observed regardless of weight status in rats. Interestingly, phoenixin’s concentration measured by an ELISA assay in the rat heart was increased after an ischemia protocol (Rocca et al., 2018). The authors speculate about a possible autocrine or paracrine regulation of cardiac function, employing the nitric oxide (NO) pathway (Rocca et al., 2018) leading to a decrease in L-type calcium channel currents (Abi-Gerges et al., 2001). Phoenixin-14 has shown both cardioprotective properties by increasing contractile function after an ischemia protocol and reducing apoptosis, both also in obese rodents, as well as negative properties such as the reduction in relaxation, which is also present in cardiac pathologies such as heart failure with normal ejection fraction (Rocca et al., 2018). Phoenixin also seems to be linked to hypertension, with a recent report showing an inverse correlation between blood pressure and serum phoenixin-14 and -20 levels as well as body weight and phoenixin levels (Akdu et al., 2022). Notably, this study investigated both male and female subjects (Akdu et al., 2022). The recently presented evidence suggesting a protective effect of phoenixin-20 against ox-LDL-induced atherosclerosis (Wei et al., 2021) together with its altered levels in arterial hypertension and its proposed cardioprotective properties could potentially make it a target for pharmaceutical treatment of hypertension or cardiac dysfunction.

#### 2.4.4 Memory and cognitive function

Phoenixin-14 was shown to improve both memory formation as well as memory retention when injected icv or infused into the hippocampus in mice (Jiang et al., 2015b). Specifically, it attenuated the memory impairment caused by amyloid-β1-42 and scopolamine (Jiang et al., 2015b), which would hint towards possible applications in the treatment of amyloid-caused dementia. This proved to be a GnRH receptor dependent effect, since it was blocked by the GnRH receptor antagonist cetrorelix (Jiang et al., 2015b). In a human *in vitro* stroke model, phoenixin-14 was shown to protect brain endothelial cells against oxygen-glucose deprivation/reoxygenation (OGD/R) injury through an attenuation of ROS and NADPH-Oxidase-1 (NOX1), while inhibiting high-mobility-group-box-1 (HMGB1) expression and simultaneously enhancing the protective molecule NO (Zhang and Li, 2020). All effects were dose-dependent and the effects of OGD/R were either fully recovered or greatly attenuated by 200 nM phoenixin-14 treatment over 24 h (Zhang and Li, 2020). The observed reduction in epithelial permeability was attributed to increased occludin expression *via* the Kruppel-like factor 2 (KLF2) pathway (Zhang and Li, 2020).

Another neuroprotective pathway was observed in a human neuronal cell culture model, where phoenixin-20 enhanced mitochondrial biogenesis through the cAMP-response-element-binding protein (CREB) pathway and an induction of peroxisome proliferator-activated receptor γ-co-activator 1α (PGC-1α), NRF-1 (nuclear respiratory factor 1) and TFAM (transcription factor A, mitochondrial) as well as an increased ATP production, supporting the survival of neurons (Yang et al., 2020). Similarly, phoenixin-20 also showed to have protective properties in an acute stroke model, where it reduced the infarcted area while also improving neurological function scores in a mouse model (Wang et al., 2022). The suggested pathway of this neuroprotective effect was also shown to be GPR173 dependent (Wang et al., 2022).

Apart from stroke models, phoenixin-14 was recently shown to be a potential therapeutic target for epilepsy, since it reduced interictal-like spikes in an epilepsy model in murine brain slices (Kalkan et al., 2021). This is especially interesting given the fact that a deletion of phoenixin’s precursor molecule was shown to induce epileptiform seizures (Kinton et al., 2002).

#### 2.4.5 Energy homeostasis—food and fluid intake

There are several studies investigating the effects of phoenixin on food and fluid intake. Phoenixin-14 injected icv lead to a significant increase in food intake, which was not observed after peripheral (intraperitoneal, ip) injection (Schalla et al., 2017). This was accompanied by an activation of nesfatin-1 neurons in the LS, SON, PVN and NTS (Friedrich et al., 2019). Animals treated with icv phoenixin siRNA did not show weight loss during an 8-day period (Yosten et al., 2013). Phoenixin plasma levels were significantly elevated during the postprandial phase in rats (Rocca et al., 2018). This, however, was not observed in obese rats that received a high-fat diet (Rocca et al., 2018). It should be mentioned that it is unclear whether the chow or the obesity caused the lower plasma levels (Rocca et al., 2018).

With regard to fluid homeostasis, phoenixin siRNA did not lead to a reduction in vasopressin in the hypothalamus (Yosten et al., 2013), while phoenixin-20 increased the release of vasopressin *in vitro* in hypothalamo-neurohypophyseal explants (Gasparini et al., 2018) as well as *in vivo* in the plasma of icv injected animals (Gasparini et al., 2018) with a peak around 20 min after injection. This effect was dose-dependent and disrupted by icv GPR173 siRNA injection, leading to the assumption that the effect is GPR173 dependent (Gasparini et al., 2018). A role in fluid and electrolyte homeostasis was suggested (Gasparini et al., 2018). Concurrently, icv injection of phoenixin-14 (Friedrich et al., 2019) and -20 have shown to increase drinking behavior in both male and female rats, which was ameliorated by pretreatment with losartan or blockage of GPR173 before phoenixin-20 application (Haddock et al., 2020). Male rats were, however, less affected by phoenixin-20 application than female rats in this experiment (Haddock et al., 2020), showing a sex-specific effect of phoenixin in non-reproductive functions. Phoenixin-20 also stimulated the release of vasopressin both *in vivo* and *in vitro* in explants containing the SON as mentioned above (Gasparini et al., 2018), which taken together with the observed blockage of phoenixin’s dipsogenic properties by losartan suggests an involvement of phoenixin in vasopressin-dependent fluid regulation.

Phoenixin mRNA expression was also shown to increase in the hypothalamus after fasting and return to normal levels within 3 h after refeeding in spotted scats (Wang et al., 2018). Since phoenixin’s precursor SMIM20 mRNA is present in preadipocytes of rats and phoenixin-14 itself is also involved in the proliferation and storage of lipids in preadipocytes (Billert et al., 2018), phoenixin is likely not only involved in food intake but also in energy storage and body mass, further supported by the previously shown correlation with BMI (Yuruyen et al., 2017). Expression of phoenixin mRNA in immortalized hypothalamic neurons is influenced by several fatty acids, namely palmitate, oleate and DHA, leading to the authors’ assumption of an involvement in nutrient sensing (McIlwraith et al., 2018). Phoenixin is also present in pancreatic alpha and beta cells, from which its secretion is induced by high glucose levels (Billert et al., 2019). In a recent report, phoenixin-14 also reduced pancreatic injury induced by streptozotocin (STZ) without affecting insulin levels (Ozdemir-Kumral et al., 2022), hinting towards a role in glucose-homeostasis through the protection of pancreatic cells. Phoenixin-14 furthermore enhanced the secretion of insulin from pancreatic INS-1E cells through modulation of cAMP/Epac signaling (Billert et al., 2019), further suggesting an involvement of phoenixin not only in appetite and food intake but also energy- and glucose-homeostasis.

Due to its highly conserved nature (Yosten et al., 2013), phoenixin-20 has also been observed to mediate food intake in fish, where its levels were decreased after fasting in several tissues, namely the brain, liver, muscle and gonads (Rajeswari et al., 2020). Interestingly, peripheral injection of phoenixin-20 did have an effect in fish, where it lead to a reduction in feeding in both sexes (Rajeswari et al., 2020) as opposed to the lack of an effect of a peripheral injection of phoenxin-14 in rodents (Schalla et al., 2017).

It should be noted, that phoenixin is mostly described as an orexigenic peptide in mammals, contrary to the observed reduction in food intake in zebrafish (Rajeswari et al., 2020), suggesting not only a sex-specific, but also a species-dependent mode of action. Although not statistically significant, SREB3 – also known as GPR173 – was elevated after fasting in the gut and liver (Rajeswari et al., 2020). In patients suffering from anorexia nervosa, phoenixin serum levels were decreased and normalized with weight gain, with body weight and phoenixin levels being positively correlated (Palasz et al., 2019). Although the causality is not yet known – whether reduced phoenixin levels possibly cause or enhance anorexia nervosa symptoms or potentially are a result of the reduced food intake – a clear connection can be observed.

## 3 Implications in stress and anxiety

Phoenixin was mostly studied in rodents, where icv injection of phoenixin-14 showed a dose dependent anxiolytic effect (Jiang et al., 2015a). This was in part attenuated by a GnRH receptor antagonist while being unaffected by vasopressin/oxytocin receptor antagonists (Jiang et al., 2015a). Direct infusion into the amygdala did not show any anxiolytic effects (Jiang et al., 2015a), leading to a proposed GnRH dependent mode of action most likely located in the anterior hypothalamic area (AHA) (Jiang et al., 2015a). Therefore, a GnRH receptor dependent and oxytocin independent anxiolytic effect has been proposed (Jiang et al., 2015a). In humans, a negative association between plasma phoenixin levels and anxiety in obese men was observed (Hofmann et al., 2017).

In addition to anxiolytic effects, phoenixin injected icv into the lateral ventricles led to a dose dependent decrease in core body temperature, which was also in some form dependent on GnRH receptor activity, since its blockage decreased phoenixin-14’s effects (Jiang et al., 2015a). The reduction in body temperature peaked 20 min after injection and lasted for roughly 1 hour (Jiang et al., 2015a).

As shown previously by our research group, both restraint stress (Friedrich et al., 2020) and immunological LPS stress (Friedrich et al., 2022) altered central phoenixin immunoreactivity. Restraint stress simulated emotional stress and caused an increase in activity and phoenixin density in several nuclei, namely the RPa, mNTS and DMN (Friedrich et al., 2020). An effect of restraint stress on phoenixin was also shown in another restraint stress study, which showed a decrease in phoenixin plasma concentration 15 min after restraint stress was administered (Schalla et al., 2020). These levels correlated with serum nesfatin-1 levels (Schalla et al., 2020).

Immunological stress exerted through an ip injection of lipopolysaccharide (LPS) resulted in a similar pattern in brain activity, namely activating the SON, CeM, RPa, mNTS and DMN and increasing phoenixin immunoreactivity (Friedrich et al., 2022). Phoenixin-20 was shown to have protective properties against LPS induced inflammation in dental pulp cells, where it reduced the release of LDH and attenuated the activity of the TLR-4, Myd88 and NF-κB pathway (Sun et al., 2020). This anti-inflammatory response employed GPR173 since silencing of GPR173 with shRNA abolished the effects (Sun et al., 2020). In another study, the authors showed an attenuation of the stress response of the endoplasmatic reticulum (ER) as a protective mechanism of phoenixin-14 against LPS-induced immunological stress in astrocytes (Wang et al., 2020). It also reduced the activation of the NLRP3 inflammasome and the abundance of ROS as well as decreased IL-1β and IL-18 production (Wang et al., 2020). The effects of phoenixin-14 on the NLRP3 expression and the reduced interleukin production were GPR173 dependent as shown by siRNA knockdown (Wang et al., 2020). Phoenixin-14 also reduced the increase of the high mobility group box 1 (HMGB1) protein (Wang et al., 2020), an inductor of NLRP3 expression (Frank et al., 2016) under conditions of inflammatory stress. This protective mechanism was also present in the OGD/R model (Zhang and Li, 2020). The same attenuation of the NLRP3 inflammasome was observed in microglial cells *in vitro* when treated with phoenixin-20 (Zeng et al., 2020), where the expression of thioredoxin-interacting protein (TxNIP), necessary for NLRP3 inflammasome activation (Nasoohi et al., 2018), was reduced by phoenixin, with phoenixin-14 and -20 showing a similar mode of action. The effects of phoenixin-20 were dependent on the sirtuin-1 (SIRT-1) pathway, since the inhibition of this pathway by nicotinamide abolished the effect (Zeng et al., 2020). Apart from neuroprotective properties, phoenixin-14 was also shown to ameliorate indomethacin-induced duodenal ulcers (Zandeh-Rahimi et al., 2022). Although in this study the model was used as a surrogate for inflammatory bowel disease, its results might hint towards effectiveness in the treatment of stress-induced ulcers as well, since its effectiveness surpassed that of a histamine-2-receptor antagonist, which is considered a treatment option in stress ulcers (Young et al., 2020; Zandeh-Rahimi et al., 2022).

The effects of phoenixin on neuron excitability in the NTS, a nucleus that both contains phoenixin and is activated by it (Prinz et al., 2017; Friedrich et al., 2022), has also been shown by a distinctive decrease in spike frequency variability when animals were – by accident – subjected to construction noise while also receiving phoenixin treatment (Grover et al., 2020). The construction noise had a similar effect as a pretreatment with corticosterone (CORT) for 22 days (Grover et al., 2020). Without the stressor present, phoenixin increased the firing frequency as well as the depolarization by 32% and 50%, respectively (Grover et al., 2020). These results suggest that phoenixin also plays a role in the reaction to external sensory stressors – noise in this case – and not only internal stressors such as endotoxin presence or emotional restraint stress.

As described above, nesfatin-1 is seemingly interconnected with phoenixin, exerting an antagonistic effect in most regards. Apart from food intake and anxiety, nesfatin-1 is also involved in various stressors such as surgical stress (Stengel et al., 2010), metabolic stress (Sun et al., 2021) and restraint stress (Sun et al., 2021). The importance of nesfatin-1 in stress is further underlined by its capability to activate the hypothalamus-pituitary-adrenal (HPA) axis and increase corticosterone levels in plasma after it was applied icv (Konczol et al., 2010). As reported previously, corticosterone treatment for 22 days abolished the effects of phoenixin on NTS neuron excitability (Grover et al., 2020), thereby suggesting an indirect link between nesfatin-1 and phoenixin in chronic stress, where an increase in corticosterone induced by nesfatin-1 signaling reduced phoenixin’s effects on neuron excitability, thereby indirectly reducing neuron excitability in the NTS, which is the “gateway” nucleus for peripheral sensory signals (Ran et al., 2022). This could be interpreted as an adaptation or desensitization towards the stressor in order to avoid an excessive stress response. A previously proposed pathway could be an activation of nesfatin-1 receptors in the PVN, a highly relevant nucleus in stress reactions which contains many CRF neurons regulating ACTH release (Vermetten and Bremner, 2002). Phoenixin depolarized magnocellular neurons in the PVN and increased firing frequency *in vitro* (Gasparini et al., 2018). The PVN was also shown by autoradiography to contain nesfatin-1 receptors (Prinz et al., 2016), leading to the assumption of an activation of nesfatin-1-receptor expressing neurons and thereby increasing the activity of the HPA axis. Since nesfatin-1 is able to cross the blood-brain barrier (Pan et al., 2007) and also elicited an increase in CRF mRNA expression in the hypothalamus and increased plasma corticosterone levels after chronic application (Ge et al., 2015), interaction of peripheral and central signaling might also play a role. To summarize this theory, stressors relayed *via* the NTS could lead to an increase in nesfatin-1 signaling, which then activates the PVN and increases corticosterone levels, which in turn over a longer period of time reduce phoenixin effects in the NTS, thereby reducing the amount of peripheral sensory stress input relayed *via* the NTS and allowing for an adaptation to the stressor.

## 4 Pharmacological properties and outlook

**FIGURE 1:**
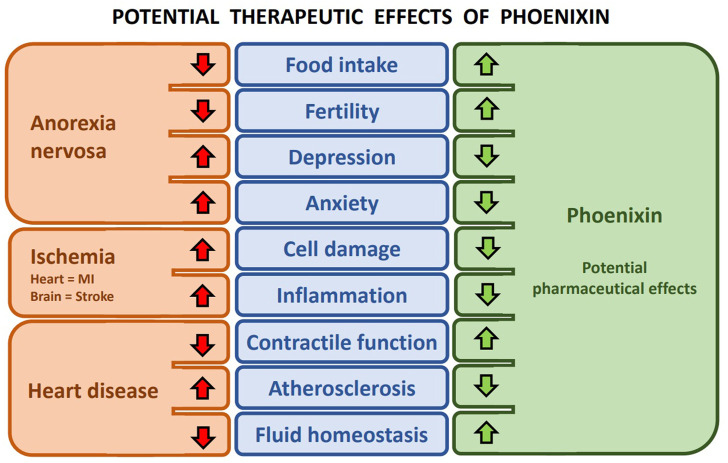
Potential therapeutic effects of phoenixin–The effects of pathologies (red arrows) on physiological functions (blue) and the associated possible therapeutic effects of phoenixin (green arrows) on those affected functions.

As of now, phoenixin is not yet in use for human pharmaceutical purposes, since research on phoenixin and its possible pharmacological properties are still in its infancy. The currently published studies do, however, provide us with some insight into its possible and probable clinical applications. The observed effects of phoenixin are mainly increased appetite (Schalla and Stengel, 2019), reduced anxiety (Jiang et al., 2015a), increased fertility (Basha et al., 2022) and cardioprotective properties (Wei et al., 2021). These fall perfectly in line with the pathologies observed in patients with anorexia nervosa, namely inhibited appetite and reduced body weight, increased parameters of anxiety and depressiveness and decreased fertility as a result of the low body weight (Figure 1) (Zipfel et al., 2015; Westmoreland et al., 2016). Anorexia nervosa is also one of the psychiatric diseases, for which both pharmacological as well as psychotherapeutic treatment still render unsatisfactory results with a high risk of relapse. Its mortality rate is the highest of all psychiatric disorders (Edakubo and Fushimi, 2020). New therapies are therefore of the utmost importance for patients suffering from anorexia nervosa.

The phoenixin pathway seems a possible target for future drug development. Its anxiolytic and orexigenic properties could be helpful in both the initial phase of treatment in order to stabilize body weight and reduce the malnutrition as well as in long-term treatment, possibly by both exerting an anxiolytic as well as an antidepressant effect. As previously reported, phoenixin serum levels were significantly lower in patients suffering from anorexia nervosa compared to healthy controls (Palasz et al., 2019). The observed normalization of phoenixin serum levels when patients gained weight and the resulting positive correlation between phoenixin and body weight normalization (Palasz et al., 2019) deliver an initial hint towards a possible role of phoenixin in the pathogenesis of anorexia nervosa.

Apart from its known effects on food intake and anxiety, a relevant information for the practicality of phoenixin as a pharmaceutical target that remains to be elucidated in order to evaluate usefulness is whether or not it can cross the blood-brain barrier. This question is not yet answered, hints in either direction exist (Schalla et al., 2017; Rocca et al., 2018). In the absence of specific active transport processes, size and polarity i.e. lipophilicity of molecules can determine its ability to cross the blood-brain barrier (Mangas-Sanjuan et al., 2010). While smaller molecules have a much better chance of passing it, larger molecules could potentially shield dipolar parts of a molecule and thereby aide its ability to cross (Warren, 2018). Threshold values for size and polarity are believed to be around <400 Da with less than eight possible hydrogen-bonding spots exposed by the protein (Pardridge, 2012). Although smaller molecules without any further modification have a better chance of passing the blood-brain barrier, neither the small phoenixin-14 molecules nor the larger SMIM20 molecules should be ruled out as possible pharmacological targets. It should be noted that the differentiation between phoenixin’s ability to penetrate into the cerebrospinal fluid and its ability to cross the blood-brain barrier is very important (Pardridge, 2012). Interestingly, icv administration of phoenixin led to a different effect than peripheral injection as described above. Besides reaching adjacent nuclei after icv injection (Yan et al., 1994), phoenixin could also be applied intranasally. Other peptides such as orexin A (Dhuria et al., 2009) as well as the GLP-1 antagonist exendin_(9–39)_ (Banks et al., 2004) were shown to be able to reach the brain this way in early animal models. The ability to avoid the blood-brain barrier this way is commonly discussed (Hanson and Frey, 2008) but not universally agreed upon (Pardridge, 2019). All of these speculations warrant further research, specifically the distribution of phoenixin after icv administration, elucidation of its ability to permeate the blood-brain barrier as well as its effects once it has crossed the blood-brain barrier.

Another aspect of phoenixin in regard to its pharmacological potential is the observed sex specific nature of its effects (McIlwraith et al., 2018; McIlwraith et al., 2019; Haddock et al., 2020). In addition, the receptor and its binding sequence have not yet been positively identified and proven, hindering the research into possible ligands and agonists as well as antagonists. Since GPR173 has recently been doubted as phoenixin’s receptor (Yanez-Guerra et al., 2022), the identification of phoenixin’s receptor is of utmost importance. Expression and coexpression studies as well as further research on the affinity of phoenixin to GPR173 as well as other GPRs could also hint towards possible side effects and interactions with other pathways and should be explored. Aside from phoenixin itself, a further elucidation of its pathway and proposed antagonist nesfatin-1 will be of importance. As of now it is unclear if phoenixin and nesfatin-1 interact directly, through their respective receptors or possibly even share binding capabilities for GPR173. Therefore, an indirect influence on the phoenixin pathway through nesfatin-1 modulation might be an option further down the line.

Importantly, it should be mentioned that all of phoenixin’s possible drug targets and pharmaceutical use cases remain highly speculative at the moment and the hypotheses discussed above should be interpreted as such. Current scientific data is by far premature for a use of phoenixin or its pathway in human medical drug treatment within the coming decade. Nonetheless, as outlined in this review, the possibilities are plentiful and from the limited research that is available, the properties of phoenixin seem promising. There is, however, much to be studied in order to gain more insight into phoenixin and develop use cases.
