# Supplementary material for: Current state of phoenixin—the implications of the pleiotropic peptide in stress and its potential as a therapeutic target
Source: Front Pharmacol. 2023 Feb 13;14:1076800. doi: 10.3389/fphar.2023.1076800 (PMC9968724; doi:10.3389/fphar.2023.1076800)
Supplement: Supplementary file 1 [file Table1.DOCX]

| **Location** | **Phoenixin** | | | **GPR173** | |
| --- | --- | --- | --- | --- | --- |
| **Type** | **Peptide** | **mRNA** | **IR** | **Peptide** | **mRNA** |
| Heart | ● | ● |  |  | ● |
| V. cava |  |  |  |  | ● |
| Aorta |  |  |  |  | ● |
| Thyroid |  |  |  |  | ● |
| Thymus | ● |  |  |  |  |
| Retina |  |  |  |  | ● |
| Eye |  |  |  |  | ● |
| GI Tract | ● |  | ● |  |  |
| Spleen | ● |  |  |  |  |
| Pancreas | ● |  | ● |  |  |
| Adrenal |  | ● |  |  |  |
| Ovary |  | ● |  | ● | ● |
| Uterus |  | ● |  |  | ● |
| Kidney |  | ● |  |  |  |
| Hypothalamus | ● | ● |  |  | ● |
| Amygdala |  | ● | ● |  | ● |
| Cerebellum |  | ● |  |  | ● |
| Pituitary |  | ● | ● |  | ● |
| Brainstem |  | ● |  |  | ● |
| Hippocampus |  |  |  |  | ● |
| Cerebral cortex |  |  |  |  | ● |
| Striatum |  |  |  |  | ● |
| Spinal chord |  |  | ● |  |  |
| BST |  |  | ● |  | ● |
| PVN |  |  | ● |  | ● |
| SON |  |  | ● |  | ● |
| Lateral hypothalamus |  |  | ● |  | ● |
| Arc |  |  | ● |  |  |
| VMH |  |  | ● |  | ● |
| Median eminence |  |  | ● |  |  |
| Zona incerta |  |  | ● |  |  |
| Perifornical area |  |  | ● |  |  |
| Dorsal hypothalamus |  |  | ● |  |  |
| vsc |  |  | ● |  |  |
| dsc |  |  | ● |  |  |
| Substantia nigra reticulata |  |  | ● |  |  |
| EW |  |  | ● |  |  |
| Rpa |  |  | ● |  |  |
| Sp5 |  |  | ● |  |  |
| mNTS |  |  | ● |  |  |
| DMN |  |  | ● |  |  |
| AP |  |  | ● |  |  |
| Lateral septum |  |  |  |  | ● |
| Piriform cortex |  |  |  |  | ● |
| Medial preoptic nucleus |  |  |  |  | ● |
| AVPN |  |  |  |  | ● |
| Dorsomedial nucleus of the hypothalamus |  |  |  |  | ● |
| Ventral premammillary tract |  |  |  |  | ● |
| Olfactory bulb |  |  |  |  | ● |
| Adipocytes |  |  |  | ● |  |

**Supplementary table 1**

Location of phoenixin vs. GPR173. IR = immunoreactivity
